# Non-ischemia Cardiomyopathy and Polycythemia Secondary to Anabolic-Androgenic Steroid Use

**DOI:** 10.7759/cureus.66850

**Published:** 2024-08-14

**Authors:** Raquel Rudy, Mustafa Basree, Aasems Jacob, Rishi Raj

**Affiliations:** 1 Hematology, University of Pikeville-Kentucky College of Osteopathic Medicine, Pikeville, USA; 2 Radiation Oncology, University of Wisconsin, Madison, USA; 3 Hematology and Oncology, University of Pikeville-Kentucky College of Osteopathic Medicine, Pikeville, USA; 4 Hematology and Oncology, Pikeville Medical Center, Pikeville, USA; 5 Endocrinology, Diabetes and Metabolism, Pikeville Medical Center, Pikeville, USA

**Keywords:** anabolic-androgenic steroid, non-ischemic cardiomyopathy (nicm), anabolic steroid abuse, secondary polycythemia, dilated cardiomyopathy, clinical hematology, drug-induced cardiomyopathy, hypertension, heart failure

## Abstract

Athletes and bodybuilders use anabolic-androgenic steroids (AAS) to increase muscle mass and enhance performance. Its use is widespread among competitive athletes in order to enhance athletic performances. However, the use of AAS has been linked to many deleterious adverse effects, including cardiomyopathy and polycythemia. We present the case of a young man in his late 20s who presented with uncontrolled hypertension and elevated hemoglobin. He was found to have a reduced left ventricular ejection fraction of 20-25%. Further workup showed dilated cardiomyopathy and low normal erythropoietin (EPO) levels. Evaluation for polycythemia vera was negative, and there was no evidence of ischemic cardiomyopathy. The patient later admitted to using injected AAS for professional bodybuilding. The coexistence of both these conditions can be challenging to diagnose and treat. While primary and secondary polycythemia can lead to hyperviscosity and result in ischemic cardiomyopathy from coronary occlusion, anabolic steroids can directly result in cardiomyopathy and polycythemia. This case points to the importance of identifying cardiomyopathy and polycythemia from illicit drug use, which can often be missed, and the workups needed to identify the etiology.

## Introduction

Anabolic-androgenic steroids (AAS) are synthetic derivatives of testosterone first synthesized in the late 1930 [1]. AAS are used to treat various conditions, predominantly male hypogonadism and chronic diseases such as cancer and renal insufficiency, among others [1]. AAS has also been misused by athletes and bodybuilders, with an estimated three million people in the United States who have used AAS to improve performance and increase muscle mass [2]. The most common age of use is between 20 and 40 years old with a higher prevalence in males and those who participate in weight lifting, martial arts, and competitive sports in addition to bodybuilding [3]. Historically, professional athletes have used AAS to enhance performance and gain a competitive advantage; however, the prevalence of this has decreased since the implementation of routine drug screens and anti-doping committees [3,4]. AAS can be taken orally or injected and are typically given in cycles, which typically last between four and 18 weeks. Multiple AAS may be used together, which is referred to as a "stack," to achieve higher doses with rapid effects [4]. The use of AAS carries various risks, even when used in therapeutic doses, such as dependence and endocrine abnormalities [5]. Supratherapeutic doses are often seen when AAS are used with performance-enhancing intent, which spans a broad spectrum of cardiovascular, reproductive, endocrine, hepatic, hematologic, and behavioral abnormalities. Increased blood pressure, lipid metabolism dysregulation, cardiac muscle remodeling, and increased erythropoiesis are some of those changes seen in AAS misusers [5].

Polycythemia is a condition with increased red blood cell (RBC) mass that can be primary or secondary. While the former is usually due to mutations in RBC progenitor cells resulting in increased production, the latter is caused by a physiologic response to tissue hypoxia leading to an increase in erythropoietin (EPO) production [6]. Those physiologic responses are often seen in patients with a history of smoking, hypoxic cardiopulmonary diseases, EPO-secreting tumors, or use of athletic performance-enhancing agents [6]. Primary polycythemia includes polycythemia vera and myeloproliferative neoplasms, which are commonly associated with arterial and venous thrombotic complications. They are nearly always accompanied by low to normal EPO levels [6]. Secondary polycythemia presents with elevated EPO levels except when secondary to autologous transfusions or the use of performance-enhancing anabolic steroids [6]. Obtaining a diagnosis can often become a quandary when patients present with cardiomyopathy and polycythemia, where identifying the primary condition is vital in determining appropriate management. A thorough history is crucial in these situations, as in our case. Here, we described the case of a young male presenting with cardiomyopathy and polycythemia secondary to AAS use.

## Case presentation

**Figure 1:**
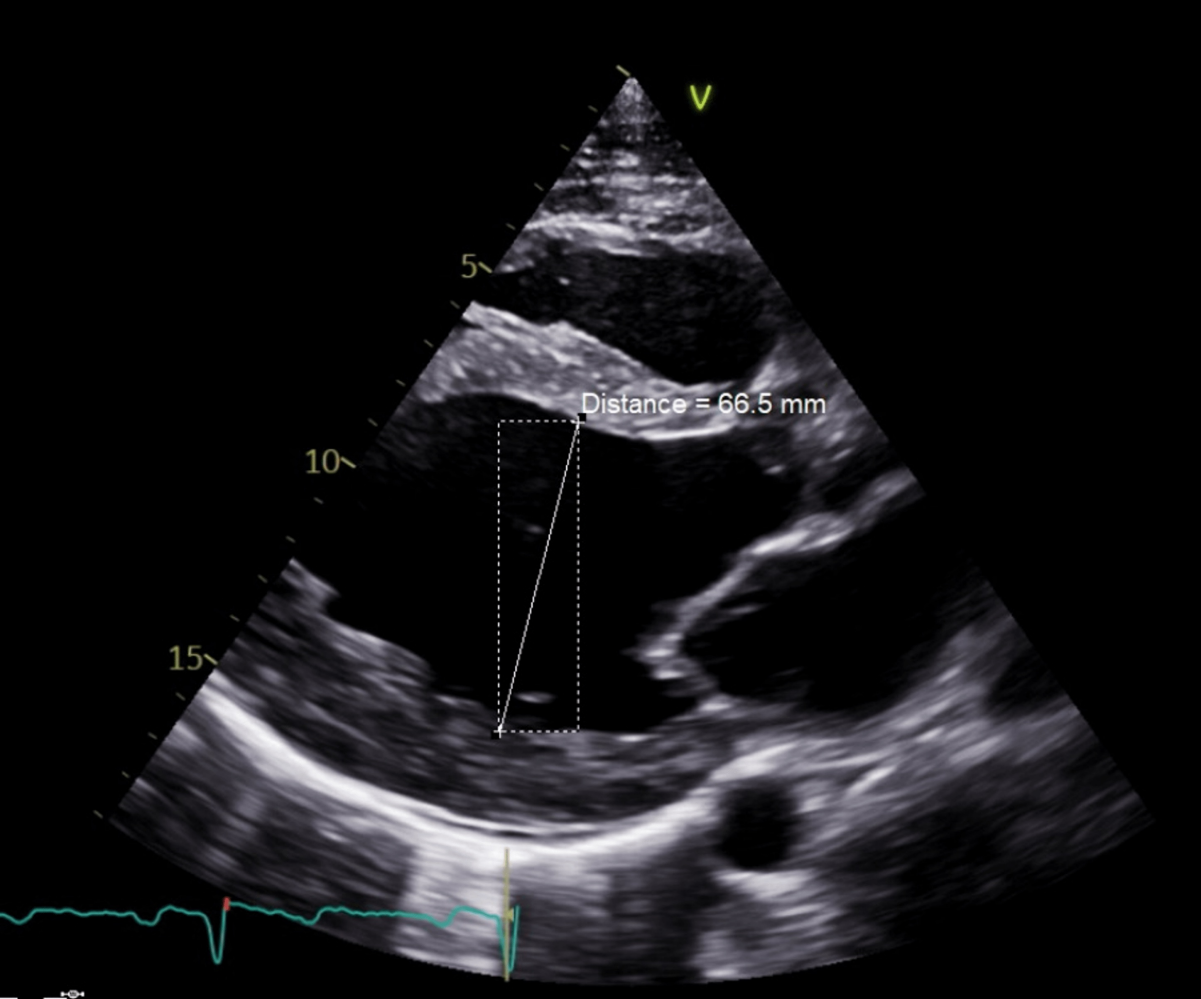
Transthoracic echocardiogram (parasagittal view) showing the left ventricular cavity measuring 66.5 mm, with an end-diastolic volume of 287 mL, suggestive of left ventricular cavity dilation.

**Figure 2:**
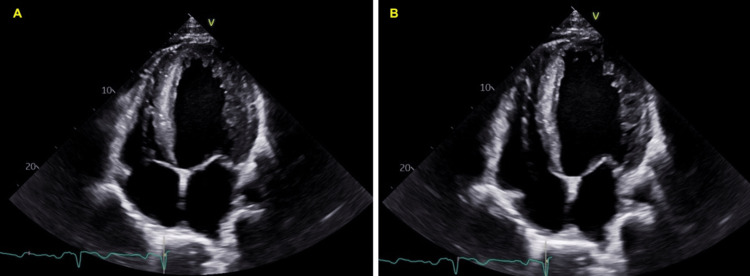
Apical four-chamber view of transthoracic echocardiogram (A) systolic phase and (B) diastolic phase with decreased left ventricular ejection fracture of 20-25%, with normal interventricular wall thickness (1 cm) and mild mitral regurgitation.

A man in his late 20s initially presented to the primary care doctor for the evaluation of exposure to coronavirus disease 2019 (COVID-19). On presentation, he denied cough, shortness of breath, palpitations, headache, or dizziness. His medical and surgical histories were negative. He consumed half a pack of beer every week and 1-2 cigarettes per week, but denied use of any illicit drugs. He was not on any prescription medication and denied using any additional supplements. He did not receive any vaccinations in the previous year. He worked in physically demanding manual labor and also worked out regularly. Relevant family history includes his father having an unknown condition requiring periodic phlebotomy. On examination, he was hypertensive at 186/105 mmHg and had a heart rate of 86 beats per minute (bpm) and a normal oxygen saturation of 98% on room air. Physical exam, including heart and lung exam, was noncontributory. There were no recent labs available for review prior to the presentation. Viral respiratory panel, including COVID-19 testing, was negative. Complete blood count (CBC) revealed elevated hemoglobin of 19.5 g/dL (normal 13.2-16.6 g/dL) and hematocrit of 59.2% (normal 38.3-48.6%). An electrocardiogram showed a first-degree atrioventricular (AV) block with a rate of 78 bpm and no ST-T wave changes. He underwent a therapeutic phlebotomy of 900 cc of blood. An outpatient transthoracic echocardiogram (TTE) two weeks later showed reduced left ventricular ejection fraction (LVEF) of 20-25% and severely dilated left ventricular cavity size (left ventricular end-diastolic volume 287 mL) with diffuse hypokinesia (Figure 1 and Figure 2). Interventricular wall (1 cm) and posterior wall thickness (1 cm) were normal. There was mild mitral regurgitation and no aortic regurgitation or left ventricular outflow tract obstruction (Figure 2). He was then referred to a hematologist and cardiologist for further evaluation and management. 

**Figure 3:**
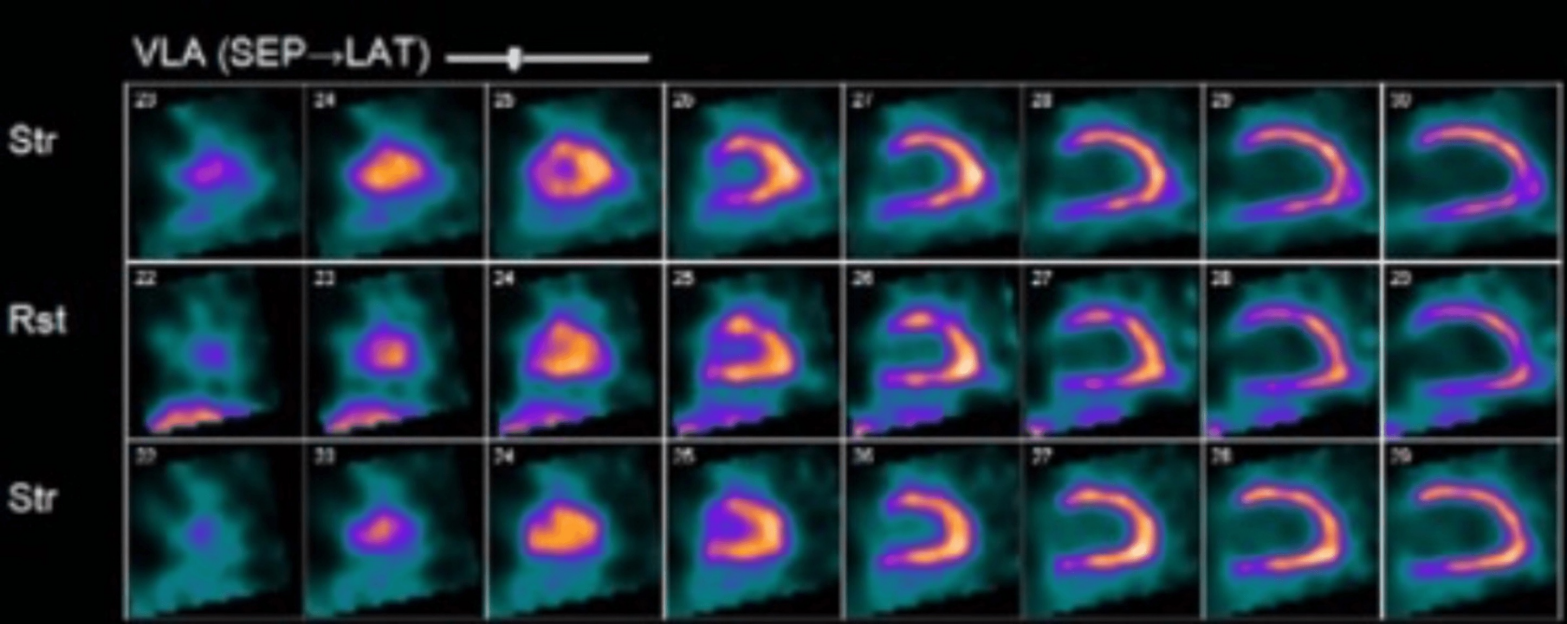
Nuclear stress test (VLA) showing a thin and dilated left ventricular wall. VLA: vertical long axis

**Figure 4:**
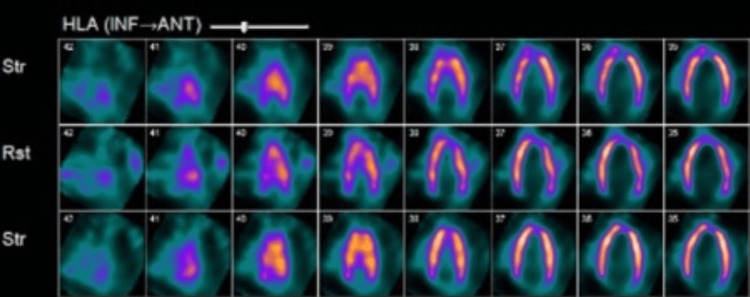
Nuclear stress test (HLA) demonstrating a thin left ventricular wall with a dilated left ventricular cavity. HLA: horizontal long axis

**Figure 5:**
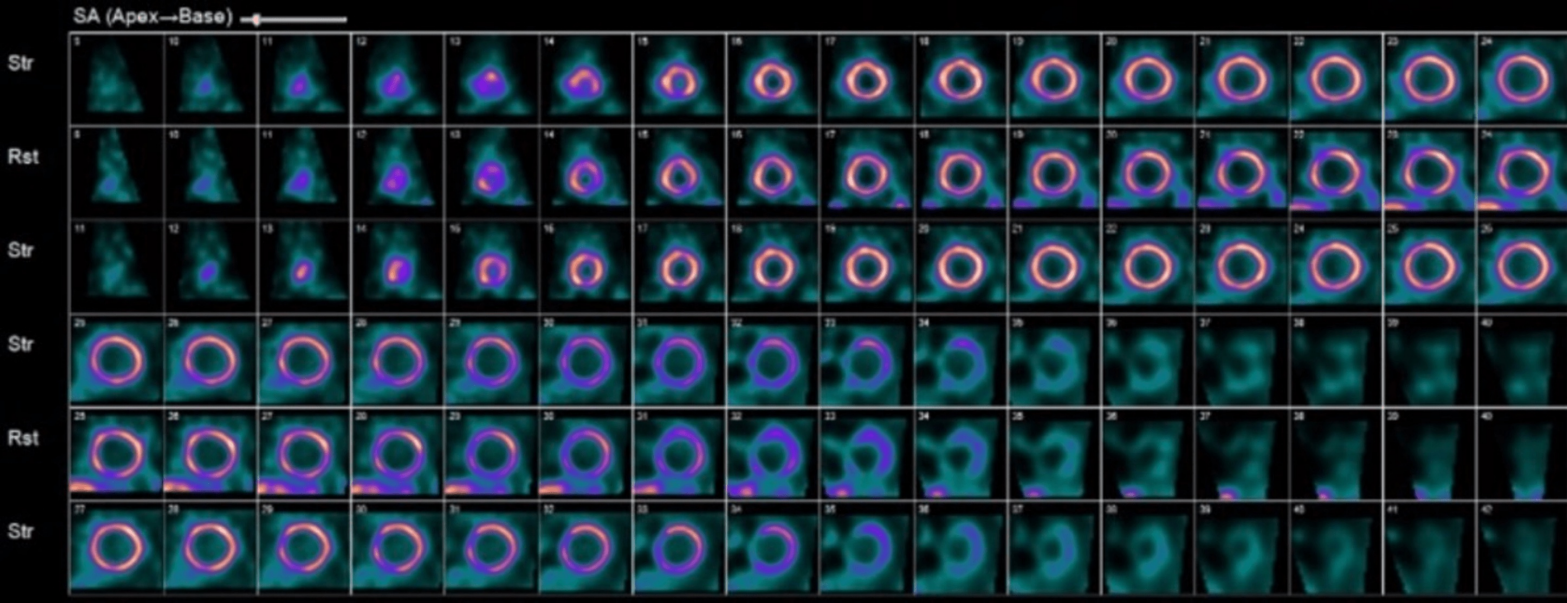
Nuclear stress test with gated tomography (parasternal short-axis view) showing homogenous tracer uptake, global left ventricular dysfunction, diffuse hypokinesis, and an LVEF of 25%. LVEF: left ventricular ejection fraction

Further workup which included a treadmill nuclear stress test using Bruce protocol completed within a week showed homogenous tracer uptake in the entire left ventricular wall which is thin and dilated, with gated tomography showing global left ventricular dysfunction, diffuse hypokinesis, and an LVEF of 25%, consistent with dilated cardiomyopathy (Figures 3-5). Since findings were not suggestive of myocardial infarction, coronary angiography was not done. Troponin-I was mildly elevated at 0.06 ng/ml (normal <0.04ng/ml) and brain natriuretic peptide (BNP) level 435 pg/ml (normal <100 pg/ml). Lipid panel was normal including total cholesterol of 153 mg/dL (normal 1-200 mg/dL), high-density lipoprotein (HDL) 43 mg/dL (>39 mg/dL), low-density lipoprotein (LDL) 90 mg/dL (0-99 mg/dL), and triglycerides 96 mg/dL (0-149 mg/dL). Lack of family history of cardiomyopathy and findings suggestive of dilated cardiomyopathy on imaging ruled out hypertrophic cardiomyopathy. Urine drug screen was negative for the use of illicit substances. Although it cannot be excluded entirely, he had no antecedent history suggestive of viral infections and took no new medications making viral cardiomyopathy or medication-related cardiomyopathy likely. 

Cardiomyopathy could have resulted in polycythemia, but this is usually associated with elevated EPO levels, ruling out primary cardiomyopathy in the differential diagnosis. Repeat hemoglobin and hematocrit after three weeks of phlebotomy showed 18.1 g/dL and 54%, respectively. EPO level at the time was 4.2 mIU/mL (normal 4-26 mIU/mL). This made polycythemia less likely secondary to heart failure. Given his positive family history of hematologic disorder requiring phlebotomy and low EPO, he was assessed for Janus kinase-2 (JAK2) V617F and exon 12-15 mutations, which were also negative. Given a negative workup and the likelihood of suspected exogenous medication or illicit substance, he admitted to using anabolic steroids regularly for professional bodybuilding for the past five years and quit recently when heart failure was diagnosed. 

He remained asymptomatic despite polycythemia and heart failure with reduced ejection fraction (HFrEF). He was started on aspirin 81 mg daily to reduce the risk of polycythemia-associated thrombosis, carvedilol 3.125 mg twice daily for hypertension, and angiotensin receptor neprilysin inhibitor (ARNI) sacubitril/valsartan 24/26 mg twice daily for both hypertension and HFrEF. A wearable defibrillator was used to reduce the risk of sudden cardiac death, considering LVEF <30%. Spironolactone was not initiated at this time as the patient remained asymptomatic and had just been initiated on therapy with a beta-blocker and ARNI. At the one-month follow-up visit, hemoglobin and hematocrit levels improved to 16.6 mg/dL and 48.3%, respectively. Repeat echocardiogram showed LVEF improving to 30-35%. No additional phlebotomy was performed given hematocrit <55%, and the patient remained asymptomatic. Sacubitril/valsartan was increased to 97/103 mg twice daily. He remained asymptomatic on the above medical regimen at the two-month follow-up visit. Repeat echocardiography after three months showed improvement in LVEF to 45-50%; hence, the wearable defibrillator was discontinued. 

## Discussion

AAS are synthetic steroidal androgens, which have been used by athletes to improve their performance and by non-athletes and bodybuilders to increase muscle mass and enhance physical attractiveness. The use of AAS is known to result in adverse effects, including coronary artery disease, cardiomyopathy, hypertension, mood disorders, hypogonadism, irregular menstruation, erythrocytosis, cholestasis, hepatic neoplasms, and potential for increased risk of prostate cancer. Contaminated products and unsafe needle practices also increase the risk of human immunodeficiency virus (HIV) and hepatitis B and C infections. 

Although AAS-induced cardiotoxicity, including myocardial infarction, cardiomyopathy, and heart failure, is well known in the literature, it continues to remain a global problem [7-9]. A meta-analysis of 187 studies showed the lifetime prevalence rate of AAS to be 1.6% among women and 6.4% among men [10]. Furthermore, around one-third of AAS users develop dependence, with roughly one million men reported in the United States alone [2]. Cardiovascular effects of AAS can be due to hyperviscosity from elevated hemoglobin, cardiac hypertrophy, or myocarditis. Moreover, long-term use of AAS results in dyslipidemia with an increase in LDL concentrations and a decrease in HDL levels, ultimately increasing the risk of coronary heart disease [5,8,11]. There are also increased myocardial oxygen requirements due to hypertrophy and the promotion of a hypercoagulable state due to enhanced atherogenesis [12,13]. Left ventricular hypertrophy and hypertension result from the activation of androgen receptors in the heart and skeletal muscle with AAS, leading to the renin-angiotensin-aldosterone system (RAAS) stimulation, which has been linked to sudden cardiac death and worsening of heart failure [14]. Since there are no specific diagnostic criteria for AAS-induced cardiomyopathy, diagnosis is based on meticulous history indicating a temporal association and ruling out other common causes of cardiomyopathy. Endomyocardial biopsy can show increased myocardial fibrosis and confirm a diagnosis [13]. The absence of cardiac magnetic resonance imaging and endocardial biopsy to confirm myocarditis is a limitation of our study. 

Testosterone and anabolic steroids stimulate erythropoiesis in a dose-dependent manner. It is assumed that AAS stimulates erythropoiesis through a direct effect on hematopoietic stem cells, including insulin growth factor-1 (IGF-1) induction through androgen receptors [15]. It has been shown to promote cell differentiation and make them more responsive to EPO [16]. Hence, androgen-induced erythropoiesis is associated chiefly with average to low EPO levels, while in contrast, secondary polycythemia from chronic hypoxia due to underlying heart disease, lung disease, and obstructive sleep apnea is associated with elevated EPO levels [17]. Hyperviscosity from increased hemoglobin concentration and increased thromboxane A2 and fibrinogen synthesis also increase the risk of thrombosis among these patients [13,14]. 

Although other case reports have discussed polycythemia and cardiomyopathy associated with AAS use, the coexistence of both is a diagnostic challenge, and very few cases have been reported in the literature [14,18,19]. Most patients presented with myocardial infarction or heart failure symptoms, including chest pain, diaphoresis, dyspnea, and orthopnea. Other symptoms included confusion, syncope, weakness, aphasia, and abdominal pain. Most reports of adverse cardiovascular effects due to anabolic steroid use have been described among young men, especially those involved in athletics and bodybuilding [20]. Typically, the etiology of cardiac conditions in patients under the age of 35 is inherited. Therefore, it is crucial to consider the possibility of anabolic steroid use as a cause of heart conditions in younger patients without any significant medical or family history. Although the adverse effects of anabolic steroid use have been well described in the literature, a recent cross-sectional, observational study revealed that most users had inadequate knowledge of AAS-associated harmful effects [21]. Increased education and awareness of the risks associated with anabolic steroid use are greatly needed, especially in schools and gym facilities. Additionally, the Guidelines for Adolescent Preventive Services (GAPS) recommends that all adolescents receive annual health guidance from a clinician to promote the avoidance of substances such as anabolic steroids, tobacco, and alcohol [22]. Treatment of cardiomyopathy should first involve cessation of offending agents and initiating heart failure treatment. Beta-blockers, angiotensin-converting enzyme inhibitors (ACEI), or ARNI have been shown for acute heart failure with reduced ejection fraction to improve symptoms and reduce mortality [23]. Secondary polycythemia is managed by treating the underlying cause, which, in our case, was related to AAS use. As discussed previously, phlebotomy can be performed if a patient is symptomatic and hematocrit is >55%; however, in patients with severe heart failure, phlebotomy should be avoided to prevent exacerbation of tissue hypoxia. An alternative is to infuse an equal volume of colloids after phlebotomy. In our experience, patients with AAS-induced polycythemia experience improvement in symptoms with phlebotomy, while patients who have secondary polycythemia from tissue hypoxia have no improvement or possibly worsening of their symptoms. Finally, low-dose aspirin should be considered to prevent the increased risk of thrombosis with polycythemia. 

## Conclusions

The use of AAS has increased among athletes and bodybuilders due to its use for performance enhancement. There are a number of well-known adverse effects associated with the use of AAS, including cardiomyopathy and polycythemia. In a young patient who presents with cardiomyopathy and polycythemia without an obvious cause, it is important to assess for risk factors, including the use of AAS. Determining the etiology of the primary condition is critical in determining the appropriate management and prevention of further adverse effects. Management in the setting of AAS-associated cardiomyopathy and polycythemia involves multidisciplinary care involving a hematologist and cardiologist in order to treat them effectively. Increased education regarding the risks of AAS use is needed, particularly in schools and gyms, to prevent AAS use and the risks associated with its use. 

## References

[1] Basaria S, Wahlstrom JT, Dobs AS (2001). Clinical review 138: anabolic-androgenic steroid therapy in the treatment of chronic diseases. J Clin Endocrinol Metab.

[2] Pope HG Jr, Wood RI, Rogol A, Nyberg F, Bowers L, Bhasin S (2014). Adverse health consequences of performance-enhancing drugs: an Endocrine Society scientific statement. Endocr Rev.

[3] de Ronde W, Smit DL (2020). Anabolic androgenic steroid abuse in young males. Endocr Connect.

[4] Mazzeo F (2018). Anabolic steroid use in sports and in physical activity: overview and analysis. Sport Mont.

[5] Albano GD, Amico F, Cocimano G (2021). Adverse effects of anabolic-androgenic steroids: a literature review. Healthcare (Basel).

[6] (2024). Diagnostic approach to the patient with erythrocytosis/polycythemia. https://www.uptodate.com/contents/diagnostic-approach-to-the-patient-with-erythrocytosis-polycythemia?search=polycythemia&source=search_result&selectedTitle=1%7E150&usage_type=default&display_rank=1.

[7] Shamloul RM, Aborayah AF, Hashad A, Abd-Allah F (2014). Anabolic steroids abuse-induced cardiomyopathy and ischaemic stroke in a young male patient. BMJ Case Rep.

[8] Tirla A, Vesa CM, Cavalu S (2021). Severe cardiac and metabolic pathology induced by steroid abuse in a young individual. Diagnostics (Basel).

[9] White M, Brennan E, Mi Ren KY, Shi M, Thakrar A (2018). Anabolic androgenic steroid use as a cause of fulminant heart failure. Can J Cardiol.

[10] Sagoe D, Molde H, Andreassen CS, Torsheim T, Pallesen S (2014). The global epidemiology of anabolic-androgenic steroid use: a meta-analysis and meta-regression analysis. Ann Epidemiol.

[11] Thompson PD, Cullinane EM, Sady SP, Chenevert C, Saritelli AL, Sady MA, Herbert PN (1989). Contrasting effects of testosterone and stanozolol on serum lipoprotein levels. JAMA.

[12] Chang S, Münster AB, Gram J, Sidelmann JJ (2018). Anabolic androgenic steroid abuse: the effects on thrombosis risk, coagulation, and fibrinolysis. Semin Thromb Hemost.

[13] Nieminen MS, Rämö MP, Viitasalo M, Heikkilä P, Karjalainen J, Mäntysaari M, Heikkilä J (1996). Serious cardiovascular side effects of large doses of anabolic steroids in weight lifters. Eur Heart J.

[14] Stergiopoulos K, Brennan JJ, Mathews R, Setaro JF, Kort S (2008). Anabolic steroids, acute myocardial infarction and polycythemia: a case report and review of the literature. Vasc Health Risk Manag.

[15] T'Sjoen GG, Beguin Y, Feyen E, Rubens R, Kaufman JM, Gooren L (2005). Influence of exogenous oestrogen or (anti-) androgen administration on soluble transferrin receptor in human plasma. J Endocrinol.

[16] Moriyama Y, Fisher JW (1975). Increase in erythroid colony formation in rabbits following the administration of testosterone. Proc Soc Exp Biol Med.

[17] Dickerman RD, Pertusi R, Miller J, Zachariah NY (1999). Androgen-induced erythrocytosis: is it erythropoietin?. Am J Hematol.

[18] Garner O, Iardino A, Ramirez A, Yakoby M (2018). Cardiomyopathy induced by anabolic-androgenic steroid abuse. BMJ Case Rep.

[19] Long N, Bassi S, Pepito D, Akhondi H (2019). Gerstmann syndrome complicating polycythemia secondary to anabolic steroid use. BMJ Case Rep.

[20] Torrisi M, Pennisi G, Russo I (2020). Sudden cardiac death in anabolic-androgenic steroid users: a literature review. Medicina (Kaunas).

[21] Althobiti SD, Alqurashi NM, Alotaibi AS, Alharthi TF, Alswat KA (2018). Prevalence, attitude, knowledge, and practice of anabolic androgenic steroid (AAS) use among gym participants. Mater Sociomed.

[22] (1997). Schools and health: our nation's investment.

[23] Yancy CW, Jessup M, Bozkurt B (2017). 2017 ACC/AHA/HFSA focused update of the 2013 ACCF/AHA guideline for the management of heart failure: a report of the American College of Cardiology/American Heart Association Task Force on Clinical Practice Guidelines and the Heart Failure Society of America. Circulation.

